# A retrospective study on sex difference in patients with urolithiasis: who is more vulnerable to chronic kidney disease?

**DOI:** 10.1186/s13293-021-00382-3

**Published:** 2021-06-07

**Authors:** Tsu-Ming Chien, Yen-Man Lu, Ching-Chia Li, Wen-Jeng Wu, Hsueh-Wei Chang, Yii-Her Chou

**Affiliations:** 1 Graduate Institute of Clinical Medicine, College of Medicine, Kaohsiung Medical University, Kaohsiung, 80756 Taiwan; 2 Department of Urology, Faculty of Medicine, College of Medicine, Kaohsiung Medical University, No.100, Tzyou 1st Road, Kaohsiung, 80756 Taiwan; 3 Department of Urology, Kaohsiung Medical University Hospital, Kaohsiung, 80756 Taiwan; 4 Department of Urology, Kaohsiung Municipal Ta-Tung Hospital, Kaohsiung, Taiwan; 5 Department of Biomedical Science and Environmental Biology, College of Life Science, Kaohsiung Medical University, Kaohsiung, 80708 Taiwan; 6 Cancer Center, Kaohsiung Medical University Hospital, Kaohsiung, 807 Taiwan; 7 Center for Cancer Research, Kaohsiung Medical University, Kaohsiung, 807 Taiwan

**Keywords:** Chronic kidney disease, Urolithiasis, Gender

## Abstract

**Background:**

Urolithiasis is considered a vital public health issue with a substantial burden on kidney function. Additionally, only few reports focused on the gender difference in patients with urolithiasis. Therefore, this study aimed to compare the clinical characteristics of sex difference and their potential risk for chronic kidney disease (CKD) in patients with urolithiasis.

**Methods:**

Patients diagnosed with stone disease from 2013 to 2018 were retrospectively reviewed and divided into two groups by gender. Clinical demographic characteristics, stone location, stone composition, urine chemistries, and renal function were investigated. Univariate and multivariate analyses were used to assess the relationship and potential risk of CKD between sex groups.

**Results:**

A total of 1802 patients were included: 1312 from men and 490 from women. Female patients had a higher rate of hypertension, diabetes, and dyslipidemia. Male patients predominantly had calcium-containing stones, especially calcium oxalate stone, uric acid stone, and struvite stone. Carbonate apatite stone was more frequently found in women. Complex surgeries such as percutaneous nephrolithotomy (PCNL) and ureteroscopic lithotripsy (URSL) were more frequently performed in women than that in men. Multivariate analysis confirmed that age > 60 years (odds ratios [ORs] = 6.36; 95% confidence interval [CI], 3.8–10.8), female sex (ORs = 5.31; 95% CI 3.3–8.4), uric acid stone (ORs = 3.55; 95% CI 2.0–6.4), hypertension (OR = 7.20; 95% CI 3.8–13.7), and diabetes (OR = 7.06; 95% CI 3.1–16.2) were independent predictors of poor prognoses in CKD.

**Conclusions:**

The female gender is significantly associated with a higher prevalence of CKD among patients with urolithiasis. Therefore, women with stone disease may need close renal function monitoring during follow-up.

## Background

Urolithiasis is not an uncommon disease worldwide and is viewed as an important public health issue with a substantial burden on people’s health and considerable national economic consequences [1, 2]. Although urolithiasis rarely carries the inevitable mortality consequences, it has a profound impact on quality of life. Urolithiasis more frequently occurs in men, but women have a higher chronic kidney disease (CKD) rate [3]. Recent results from the National Health and Nutrition Examination Survey (NHANES) showed that the prevalence of stone disease in men and women was 11.9% and 9.4%, respectively, in the 2017–2018 cycle [4]. The prevalence of kidney stones steadily increased among women (6.5% in the 2007–2008 cycle to 9.4% in the 2017–2018 cycle) but not among men [4]. The gender gap in urolithiasis prevalence appears to be closing in the past decade, particularly among women younger than 60 years.

Some interesting studies have analyzed the impact of sex hormones on stone diseases [5–7]. Low testosterone levels were associated with lower odds of kidney stone diseases. Similar associations were found after adjusting for obesity, diabetes, dyslipidemia, race/ethnicity, and age [5]. In women, postmenopausal status, whether natural- or surgical-related, had a higher risk of incident kidney stones. According to a study with postmenopausal participants, the incidence of stone events was 1.4% [6]. The kidney stone rate was also lower in patients on hormonal therapy in the current or previously used population [6]. Another recent study showed no apparent association between testosterone and estradiol levels on kidney stones after adjusting for age, race, body mass index (BMI), and medical comorbidities [7]. Reports [8, 9] also showed the impact of gender on stone composition. Women have reported a higher prevalence of apatite and struvite stones, whereas men have a higher prevalence of calcium oxalate and uric acid stones [8, 9]. Women were particularly more likely than men to have infection stones. This may be due to women stone formers are at increased risk of urinary tract infection, which in turn, could raise urinary pH from infection with organisms that contain urease and favor infection stone accumulation [8]. Significant gender differences should be considered when making therapeutic options to prevent stone disease recurrences. A recent review article [10–12] concluded that urolithiasis may cause CKD, and declines in renal function generally occur in patients with preexisting CKD or a large stone burden requiring complex surgery. Moreover, even after resolving the stone disease, patients may continue to suffer from higher cystatin C levels and non-albumin proteinuria that may cause CKD in stone formers [13]. To date, our understanding on the pathophysiologic mechanisms accounting for the decrease in renal outcomes and urolithiasis remains limited. Furthermore, few reports focused on the relationship among gender, stone position, and renal function. Therefore, this study primarily aimed to compare the clinical characteristics of sex difference and their potential risk for CKD in patients with urolithiasis.

## Methods

This study was approved by the Institutional Review Board of the Kaohsiung Medical University Hospital (KMUHIRB-E(II)-20180159). Our hospital database contained records of patients with urolithiasis from March 2013 to June 2018. Patients were divided into two groups by gender. All patients had radiological evidence of urinary stones. Clinical data were retrospectively collected. Renal function was evaluated with estimated glomerular filtration rate (eGFR) using the Chronic Kidney Disease Epidemiology Collaboration (CKD-EPI) creatinine-based formula [14]. Pre- and postoperative follow-up renal functions were recorded during the same hospitalization. Post-diagnosis follow-up was also evaluated using the medical record. CKD was defined as an *eGFR* of < 60 ml/min/1.73 m^2^. Patients with solitary kidneys, congenital renal anomalies, severe urinary obstruction, and autosomal dominant polycystic kidney disease were excluded. Stone compositions were analyzed using the Fourier transform infrared spectroscopy. Images were analyzed and reported separately by two different doctors. Our inclusion criteria for hypertension were defined by two criteria: (1) ICD-9-CM diagnostic codes 401 to 405 and (2) hypertension medication use (angiotensin-converting enzyme inhibitors, angiotensin II receptor blockers, calcium channel blockers, beta-blockers, and diuretics). Patients with diabetes mellitus were also defined by (1) ICD-9-CM diagnostic codes 250 and (2) diabetes mellitus medication. Patients with hyperlipidemia were ICD-9-CM diagnostic codes 272 with hyperlipidemia medication.

## Statistical analysis

All values are expressed as a mean ± standard deviation (SD) and rate. Differences between categorical parameters were assessed using a χ^2^ or Fisher’s exact test, as appropriate. Continuous parameters were assessed using a *t*-test or Mann–Whitney–Wilcoxon test. Risk factors for CKD were derived using univariate testing. Only variables with *p <* 0.05 were considered for the model. Once these potential risk factors have been identified, a multivariate stepwise logistic regression analysis was used to identify independent prognostic factors. Clinical data were separately analyzed in the univariate analysis. Significant factors were used for multivariate analysis. Independent prognostic factors in the final model are presented with odds ratios (ORs) to predict the magnitude of the influence of the risk factor on developing CKD when present, as compared with its absence. Statistical significance was set at *p* < 0.05. SPSS 20.0J (SPSS Inc., Chicago, IL, USA) was used for all statistical analyses.

## Results

**Fig. 1:**
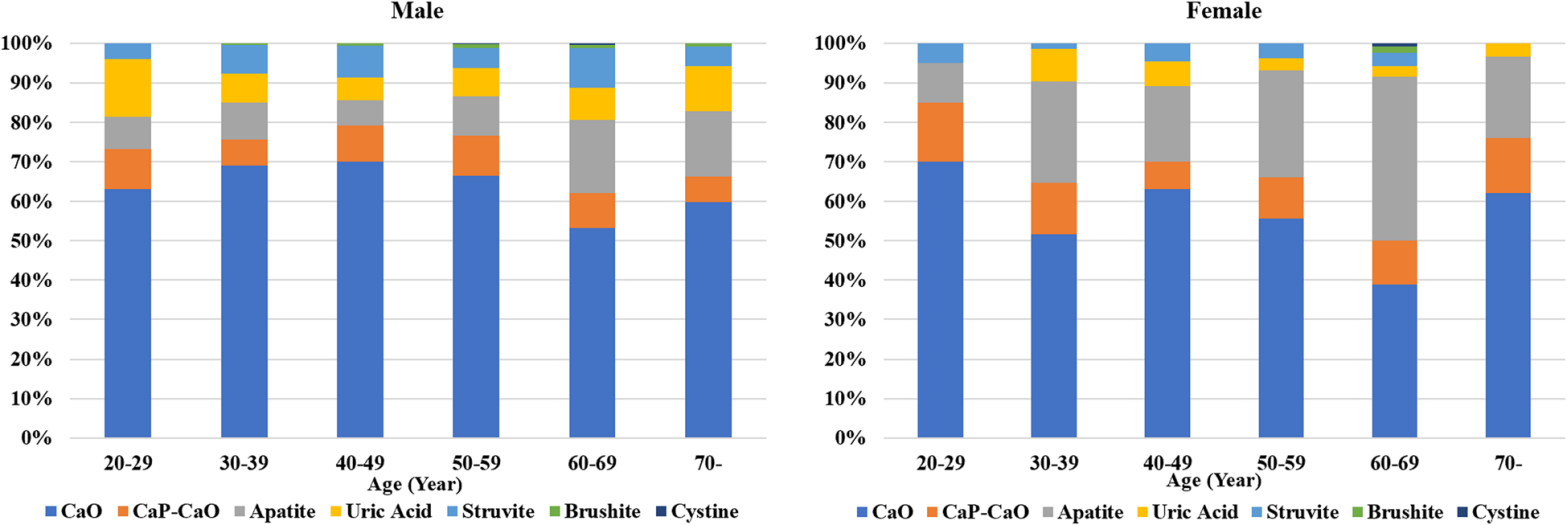
Proportions of stone compositions in different age groups and genders

We included 1802 patients between March 2013 and June 2018: 1312 from men and 490 from women. The mean age was 54.6 ± 13.6 years, and 35.2% of patients aged > 60 years. About 38.3% of patients had hypertension, 15.5% had diabetes, 8.3% had a history of dyslipidemia, 20.4% had a history of gout, and 14.8% had hyperuricemia. Men had a higher rate of gout, whereas women had a higher rate of hypertension, diabetes, and dyslipidemia (Table 1). As for the stone composition, male predominantly had calcium-containing stones, especially calcium oxalate stone, uric acid stone, and struvite stone. Carbonate apatite stone was more frequently found in women. Figure 1 shows proportions of stone compositions in different age groups and genders. Percutaneous nephrolithotomy (PCNL) and ureteroscopic lithotripsy (URSL) were more frequently performed in women than in men. Doctors performed more cystolithotripsy in men (Table 1). The average pre- and postoperative eGFRs were 85.2 ± 18.7 and 86.9 ± 16.5 mL/min/1.73 m^2^, respectively (Table 1). After an average of 3-year long-term follow-up, elderly patients (age > 60 years), women, and patients with hypertension, diabetes, uric acid stone, and lower urinary PH value were at risk of poor renal function (Table 2). Patients who underwent PCNL also had a poor kidney function prognosis (Table 2). Univariate analysis showed that age > 60 years, female sex, hypertension, diabetes, uric acid stone, and PCNL operation were risk factors for CKD. Multivariate analysis confirmed that age >60 years (ORs = 6.36; 95% CI 3.8–10.8), female sex (ORs = 5.31; 95% CI 3.3–8.4), uric acid stone (ORs = 3.55; 95% CI 2.0–6.4), hypertension (OR = 7.20; 95% CI 3.8–13.7), and diabetes (OR = 7.06; 95% CI 3.1–16.2) were independent predictors of poor prognoses in CKD (Table 3).

**Table 1:** Basic characteristics according to gender (N = 1802)

Variables	Total (N = 1802)		Men (N = 1312)		Women (N = 490)		*p*-value
	N	(%)	N	(%)	N	(%)	
Age (years)							
> 50	1158	(64.2)	828	(45.9)	330	(67.3)	< 0.001
> 60	634	(35.2)	464	(35.4)	170	(34.7)	0.790
Comorbidities							
Hypertension	691	(38.3)	500	(38.1)	191	(39.0)	0.735
Diabetes	280	(15.5)	151	(11.5)	129	(26.3)	< 0.001
Dyslipidemia	150	(8.3)	93	(7.1)	57	(11.6)	0.002
Gout	367	(20.4)	293	(22.5)	74	(15.1)	0.001
Hyperuricemia	267	(14.8)	197	(15.0)	70	(14.3)	0.698
Obesity (BMI > 27)	446	(24.8)	322	(24.5)	124	(25.3)	0.738
Stone composition							
CaO	1109	(61.5)	844	(64.3)	265	(54.1)	< 0.001
CaO-CaP	165	(9.2)	114	(8.7)	51	(10.4)	0.260
Apatite	280	(15.5)	147	(11.2)	133	(27.1)	< 0.001
Brushite	11	(0.6)	9	(0.7)	2	(0.4)	0.501
Uric acid	126	(7.0)	105	(8.0)	21	(4.3)	0.006
Struvite	108	(6.0)	91	(7.0)	17	(3.4)	0.005
Cystine	3	(0.2)	2	(0.2)	1	(0.2)	0.811
Surgery of stone acquisition							
PCNL	349	(19.4)	237	(18.0)	112	(22.8)	0.022
URSL	846	(46.9)	573	(43.7)	273	(55.7)	< 0.001
Cystolithotripsy	374	(20.8)	335	(25.5)	39	(8.0)	< 0.001
Self-voiding after ESWL	181	(10.0)	124	(9.5)	57	(11.6)	0.170
Self-voiding	52	(2.9)	33	(2.5)	19	(3.8)	0.124

*BMI*, body mass index; *CaO*, calcium oxalate; *CaP*, calcium phosphate; *PCNL*, percutaneous nephrolithotomy; *URSL*, ureteroscopic lithotripsy; *ESWL*, extracorporeal shock wave lithotripsy; *SD*, standard deviation; *SG*, specific gravity; *eGFR*, estimated glomerular filtration rate

**Table 2:** Basic characteristics according to long-term renal function

Variables	eGFR < 60 (N = 94)		eGFR ≥ 60 (N = 1708)		*p*-value
	N	(%)	N	(%)	
Age > 60	74	(78.7)	560	(32.8)	< 0.001
Female gender	59	(62.8)	431	(25.2)	< 0.001
Comorbidities					
Hypertension	51	(54.2)	640	(37.4)	< 0.001
Diabetes	42	(44.7)	238	(13.9)	< 0.001
Dyslipidemia	8	(8.5)	142	(8.3)	0.946
Gout	19	(20.2)	348	(20.5)	0.952
Hyperuricemia	19	(20.2)	248	(14.5)	0.130
Obesity (BMI > 27)	29	(30.9)	417	(24.4)	0.159
Stone composition					
Uric acid	21	(22.3)	138	(8.1)	< 0.001
Surgery of stone acquisition					
PCNL	27	(28.7)	322	(18.9)	0.019
URSL	52	(55.3)	794	(46.4)	0.095
Cystolithotripsy	6	(6.4)	368	(21.5)	< 0.001
Self-voiding after ESWL	1	(1.0)	180	(10.5)	0.003

*BMI*, body mass index; *PCNL*, percutaneous nephrolithotomy; *URSL*, ureteroscopic lithotripsy; *SD*, standard deviation, *SG*, specific gravity

**Table 3:** Independent risk factors for poor kidney function

Variables	Odds ratio	95% CI
Age (year)		
> 60	6.36	3.8–10.8
Gender		
Female/male	5.31	3.3–8.4
Stone composition		
Uric acid/nonuric acid	3.55	2.0–6.4
Comorbidities		
Hypertension	7.20	3.8–13.7
Diabetes	7.06	3.1–16.2

## Discussion

Urolithiasis has been reported as a male-dominant disease. Lin et al. [15] using the 2010 National Health Insurance report in Taiwan reported the urolithiasis (ICD-9 codes 592 and 594) male-to-female ratio in emergency visits is higher than the male -to-female ratio in non-emergency visits (2.61 in emergency vs. 1.75 in in- and out-patient clinics). We previously showed a 2.93 male-to-female ratio a decade ago [16], whereas in this study, we reported a 2.67 male-to-female ratio, indicating an increasing prevalence of stone disease among women. The gender gap in urolithiasis prevalence appears to be closing in the past decade. Several possible reasons can explain the trend of stone disease among women. Previous NHANES data show that obesity and metabolic syndrome rates significantly increased among American women [17]. Obesity also increases the risk of diabetes, hypertension, and dyslipidemia. These comorbidities had been considered to be related to stone formation [18]. Adults with high BMI not only elicit specific changes in systemic effects of the body but also influence specific organs, which may ultimately increase the stone formation [18]. A current study [19] in Taiwan showed the prevalence trend of overweight and obesity (BMI ≥ 24 kg/m^2^) has increased and remained stable. However, the prevalence trend of obesity (BMI ≥ 27 kg/m^2^) was continuously increasing. Moreover, a noticeable increase in the morbid obesity (BMI ≥ 35 kg/m^2^) prevalence was observed during the same period, from 0.4 to 1.4%. However, the prevalence of overweight decreased from 25.5 to 21.3%. These findings suggest that although the prevalence stabilized for overweight and obesity as a whole, the prevalence of obesity including “morbid obesity” is dramatically increasing. Another important reason is the prevalence of diabetes has been increasing with female preponderance over the past decades in Taiwan [20]. This may also explain the decreasing male-to-female ratio. In our study cohort, we also noted the prevalence of diabetes is higher in the female population. The NHANES study showed stone rates are similar between men and women due to the significant rising of obesity and metabolic syndrome rates among US women [4]. According to the previous report [21] analyzing diet and health trends in Taiwan, men eat more high-protein meat and high-sodium foods than women. Although the metabolic syndrome rate is increasing in both genders, we believe dietary habits and daily lifestyle still play the most important role in stone formation. Previous studies [5–7, 22] also focused on the relationship between sex hormones and physiologic changes affecting stone formation. Postmenopausal status, either natural or surgical menopause, is associated with a higher risk of kidney stone incidence [6]. Estrogen treatment is reported to decrease the risk of stone recurrence in postmenopausal women by decreasing urinary calcium and calcium oxalate saturation [22]. The mean age at menopause was 50.2 years in Taiwanese women [23]. We find that women in this series predominantly aged > 50 years compared to men (67.3% and 45.9%, *p* < 0.001, respectively). They may currently under the postmenopausal stage. This matching menopausal status for women may also explain the increase of female stone disease rate.

Urolithiasis has been associated with several serious outcomes including the development of renal function deterioration and even end-stage renal disease [3, 10–13]. One meta-analysis showed that a history of kidney urolithiasis was associated with an increased adjusted risk estimate for CKD (risk ratio, 1.47; 95% CI [1.23–1.76]) based on analyzing seven studies [11]. A large study with a mean follow-up of 12 years showed recurrent symptomatic kidney stone formers were at a higher risk for ESRD compared with non-stone formers both before and after adjustment for other comorbid conditions [12]. They concluded that the stone events are associated with kidney injury. A recent study [24] assessed whether urinary oxalate excretion is a risk factor for more rapid CKD progression toward kidney failure. They found higher oxalate excretion was independently associated with greater risks of both CKD progression and ESRD, and results were similar when treating death as a competing event. A higher urinary oxalate level is known to be associated not only with nephrocalcinosis and kidney oxalate stones but also progressive renal function deterioration in patients with enteric hyperoxaluria and primary hyperoxaluria [25]. Our previous report [3] showed patients with uric acid stones had higher age (*p* < 0.001), much lower urine pH (*p* < 0.001), and higher serum uric acid level (*p* = 0.002). Remarkably, those with uric acid stones had worse GFR than those with nonuric acid stones. We concluded that uric acid stones are associated with a higher prevalence of CKD and especially in female patients with uric acid stones.

Among women patients, there were 82 (16.5%) patients with fever or pyuria records. Only 68 (5.1%) of men had the same symptoms. The prevalence of infection stone, composed of struvite and/or apatite, among women in our study was 1.7 times that of in men (30.6% vs. 18.1%, *p* < 0.001). Women are prone to suffer from urinary tract infection, which will lead to the elevation of urinary pH level and promote the growth of urease-producing organisms [8, 26]. In our institute, PCNL was more frequently performed in the female population, which oftentimes suffered from more complex stones than the male population. In other words, female patients who underwent PCNL are thought to have more severe renal stones than those who did not. In addition, due to anatomical differences, men are more likely to suffer from benign prostatic hyperplasia and intravesical prostatic protrusion, which may subsequently obstruct the urethra, resulting in bladder stone formation [27]. Our result also showed that cystolithotripsy was more frequent in men. There were 52 (3%) patients who passed the stones spontaneously with conservative therapy and 181 (10%) patients passed the stones after ESWL treatment in the current study. In fact, lots of patients had been managed conservatively or with medical expulsive therapy (MET) and the stones had been passed spontaneously. MET is recommended for stones when there is no indication for interventional treatment. A recent study [28] demonstrated that there were no associations between gender on stone spontaneous passage. The same trend was observed in our study.

## Conclusions

We concluded that women are more prone to suffer from CKD because of multiple factors. First, they often suffered from urinary tract infection and subsequent stone formation. Second, the influence of postmenopausal status is associated with a higher risk of incident kidney stones. Third, more complicated stone needs more aggressive operation such as PCNL in women. We believed that women with urolithiasis are vulnerable to CKDs and may need close renal function monitoring during follow-up.

This study has several limitations. First, this was a retrospective analysis. Consistent with a majority of retrospective studies, data may be incomplete, missing, or inaccurate. Second, a relatively lower proportion of patients with renal stones who could be treated conservatively and with different stone burdens may have diverse effects on subsequent CKD. The database did not record stone size and numbers of episodes; therefore, we did not analyze their impact on CKD. Third, other predisposing factors, such as smoking, family history, and dietary habits, were not adjusted in our study. Fourth, selection bias may occur during the identification of the study population. Fifth, the serum creatinine was not determined using the standard isotope dilution liquid chromatography–mass spectrometry (IDLCMS) method; thus, interference may occur in plasma creatinine assays. Sixth, our results showed all men had lower BMI values than those observed in other reports [19]. A higher BMI value may influence the current results. Despite these limitations, this study is based on one of the largest databases of stones worldwide.

The difference between men and women with urolithiasis is clear. Although the incidence of urolithiasis is higher in men, the male-to-female ratio is now closer. Female patients are vulnerable to CKDs for multiple reasons. First, the incidence of diabetes mellitus is higher in the female population. Second, women had a higher infectious stone rate, which may deteriorate the urologic system. Third, a more invasive operative method can also damage the kidney. Therefore, women with urolithiasis may need close renal function monitoring during follow-up.

## Data Availability

The datasets used and/or analyzed during the current study are available from the corresponding author on reasonable request.
